# Supplementary material for: A class of hydrazones are active against non-replicating Mycobacterium tuberculosis
Source: PLoS One. 2018 Oct 17;13(10):e0198059. doi: 10.1371/journal.pone.0198059 (PMC6192558; doi:10.1371/journal.pone.0198059)
Supplement: S1 Fig — (DOCX) [file pone.0198059.s001.docx]

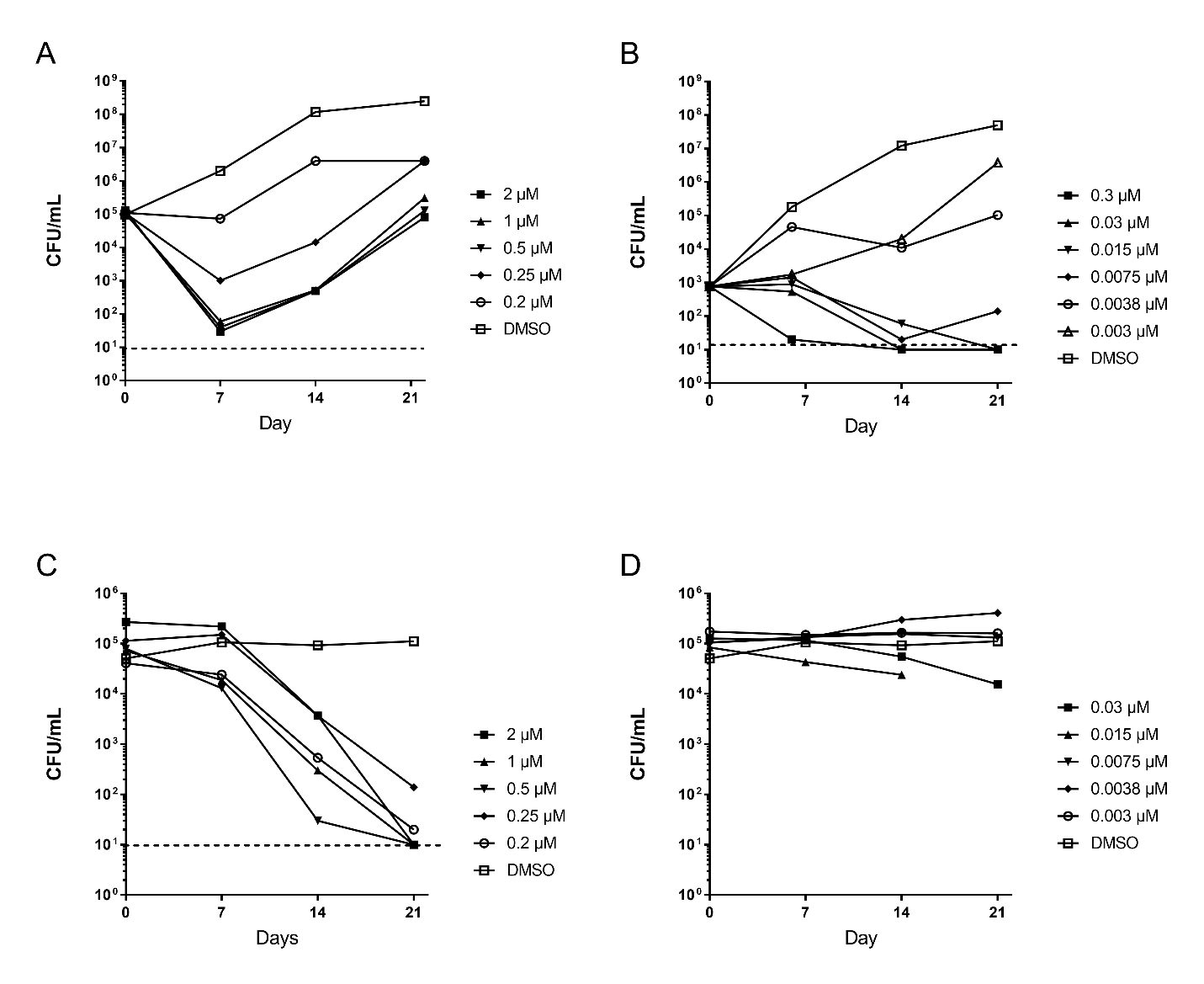


**Figure S1. Kill kinetics of isoniazid and rifampicin against replicating and non-replicating *M. tuberculosis***. Bacterial viability under replicating conditions in the presence of (A) isoniazid and (B) rifampicin was determined by counting CFU every 7 days over a 21-day period. Bacterial viability was also determined under non-replicating conditions after starving the bacteria in PBS for 2 weeks before the addition of (C) isoniazid and (D) rifampicin. The dashed line represents the lower limit of detection.
