# Supplementary material for: A class of hydrazones are active against non-replicating Mycobacterium tuberculosis
Source: PLoS One. 2018 Oct 17;13(10):e0198059. doi: 10.1371/journal.pone.0198059 (PMC6192558; doi:10.1371/journal.pone.0198059)
Supplement: S1 Table — (DOCX) [file pone.0198059.s002.docx]

**Table S1. In vitro properties of representative hydrazone compounds**

^a^The results are the average ± standard deviation from a minimum of 2 experiments

^b^MICs for wt, LepB-UE and cytotoxicity were determined previously [37]
